# Facile Fabrication of Three-Dimensional Fusiform-Like α-Fe_2_O_3_ for Enhanced Photocatalytic Performance

**DOI:** 10.3390/nano11102650

**Published:** 2021-10-09

**Authors:** Moyan Li, Hongjin Liu, Shaozhi Pang, Pengwei Yan, Mingyang Liu, Minghui Ding, Bin Zhang

**Affiliations:** 1 College of Material Science and Chemical Engineering, Harbin Engineering University, Harbin 150001, China; lmy604333@163.com (M.L.); b9838@126.com (H.L.); 18332768710@163.com (S.P.); yanpengwei@hrbeu.edu.cn (P.Y.); zhangbin_hipc@126.com (B.Z.); 2 Key Laboratory of Super Light Material and Surface Technology, Ministry of Education, Harbin Engineering University, Harbin 150001, China; 3 Institute of Surface/Interface Science and Technology, Harbin Engineering University, Harbin 150001, China

**Keywords:** α-Fe_2_O_3_ nanorods, photocatalysis, visible-light, decolorization methylene blue

## Abstract

α-Fe_2_O_3_ fusiform nanorods were prepared by a simple hydrothermal method employing the mixture of FeCl_3_·6H_2_O and urea as raw materials. The samples were examined by X-ray diffraction (XRD), high-resolution transmission electron microscopy (HRTEM), scanning electron microscopy (SEM), Fourier transform infrared (FTIR) spectroscopy and UV–vis diffuse reflectance spectra (UV–DRS). Its visible-light photocatalytic performances were evaluated by photocatalytic decolorization methylene blue (MB) in visible light irradiation. It was found that pure phase α-Fe_2_O_3_ nanorods with a length of about 125 nm and a diameter of 50 nm were successfully synthesized. The photocatalytic decolorization of MB results indicated that α-Fe_2_O_3_ nanorods showed higher photocatalytic activity than that of commercial Fe_2_O_3_ nanoparticles—these are attributed to its unique three-dimensional structure and lower electron-hole recombination rate.

## 1. Introduction

Environmental pollution has severely threatened human survival and prevented social development. However, semiconductor photocatalysis is regarded as a latent approach to solving current environmental issues [1,2,3]. Recently, the use of visible light and semiconductor photocatalysts to promote the degradation of environmental pollutants has attracted more and more attention [4,5]. In addition, available semiconductor photocatalysts (such as TiO_2_ and ZnO) are usually limited by either low efficiency in utilizing visible light or a high charge recombination rate. Hence, alternative strategies have been put forward to enhance their photocatalytic activity under visible light.

Among various metal oxide nanostructures, the scientific community has paid more attention to three-dimensional iron oxide and hydroxyl oxide nanostructures due to their inherent magnetic, morphological and phase-dependent features; they are applied in many fields such as biomedical treatment, water treatment and gas sensors. Hematite (α-Fe_2_O_3_) is considered to be one of the main forms of pure phase iron oxide, which is able to maintain the highest thermodynamic stability possible; thus it is usually used as a sensitizer for wide-bandgap semiconductors [6,7,8,9,10,11]. α-Fe_2_O_3_ has aroused great attention as a consequence of its abundant availability, environmental compatibility and very stable corundum structure [5,12,13]. However, its low conductivity, short carrier diffusion length and relatively high potential, limit the saturation current and current development potential [14]. α-Fe_2_O_3_ is prepared by methods such as chemical vapor deposition (CVD) [15,16,17], spray pyrolysis [18], hydrothermal methods [19], and precipitation [20]. Shape, size, surface structure and microstructure are the main factors which affect the chemical and physical properties of nanomaterials. Recently, different morphology types of α-Fe_2_O_3_ have been extensively studied. For instance, nanocrystals [21,22], polyhedral nanoparticles [23], nanorods [24], nanoribbons [25], nanotubes [26], nanostructured microspheres [27,28], hollow nanostructures [29,30] and nanoplates [31] are used to heighten the photocatalytic performance of α-Fe_2_O_3_. Cha et al. reported on the synthesis of α-Fe_2_O_3_ nanorods with efficient photocatalytic and magnetic properties [32]. Hao et al. synthesized single-crystalline α-Fe_2_O_3_ nanoplates, exhibiting excellent photocatalytic properties towards RhB and weak ferromagnetic behavior [33]. Chen et al. synthesized α-Fe_2_O_3_ crystals with nanoparticle, nanotube, and nanorod-like morphologies by employing a facile hydrothermal method and examined their photocatalytic activity [34]. However, there are few reports on the use of hematite with special spindle morphology as photocatalysts. Liu et al. synthesized porous fusiform-Fe_2_O_3_ (hematite) by hydrothermal synthesis assisted by a simple surfactant sodium dodecyl sulfate (SDS) [35].

Herein, we report a facile route to prepare α-Fe_2_O_3_ nanorods. The spindle β-FeOOH nanorods are firstly obtained via a water bath treatment of aqueous solution containing FeCl_3_·6H_2_O, urea and polyethylene glycol-2000. α-Fe_2_O_3_ nanorods are prepared by the calcination of β-FeOOH at 400 °C for 2 h and their photocatalytic activity is explored by degradation of the pollutant methylene blue (MB). Compared to commercial Fe_2_O_3_ nanoparticles, the α-Fe_2_O_3_ nanorods showed higher photocatalytic properties towards MB in visible light irradiation.

## 2. Experimental Section

### 2.1. Synthesis of α-Fe_2_O_3_ Nanorods

In this work, a facile hydrothermal method was used to obtain α-Fe_2_O_3_ nanorods_._ In a typical procedure, 4 g of FeCl_3_·6H_2_O, 1 g of urea and 2 g of polyethylene glycol-2000 were dissolved in 70 mL distilled water under vigorous stirring. After stirring, the resulting mixture was heated to 85 °C in a water bath for 2 h. Then, the mixture was separated by means of a centrifuge at 8000 rpm/min and washed sequentially with distilled water and ethanol repeatedly. The β-FeOOH precursor (P85) was obtained and sintered at 400 °C for 2 h in the air in a pipe furnace; then it was cooled down to obtain the final α-Fe_2_O_3_ nanorod (P85-1) sample.

### 2.2. Characterization

The phase composition of the samples was characterized by means of X-ray diffraction (XRD) (X’Pert Pro, PANalytical) operaing at 40 kV and 40 mA with Cu-K_α_ radiation (λ = 1.5406 Å). The morphology and structure of as-prepared samples were observed by HRTEM (JEM-2100) with an acceleration voltage of 200 kV. Carbon-coated copper grids were used as the sample holders. SEM was carried out using a Hitachi S-4800 instrument operating at 5 kV. FT-IR of the samples were collected with a PE Spectrum One B IR spectrometer. UV-DRS were determined by a UV-vis spectrophotometer (Shimadzu UV-2550).

### 2.3. Photocatalytic Experiments

The photo degradation experiments were performed in a quartz reactor (using a small magneton for stirring) containing 40 mL (10 mg/L) of MB solution and 0.1 g of catalyst. During the process of photocatalysis, all other lights were insulated. The high-pressure Xenon lamp (150 W, GYZ220, China) was used as a visible-light source, which was placed at about 10cm from the reactor. A 410 nm cut off filter was placed above the reactor to cut off UV light; the average light intensity was 50 mW/cm^2^. Prior to irradiation, the suspension was kept in the dark under stirring for 60 min in order to ensure the establishing of an adsorption/desorption equilibrium. At given time intervals, 4mL of aliquots were collected from the suspension and immediately centrifuged and analyzed by means of recording variations of the maximum absorption band (664 nm) of MB using a UV-visible spectrophotometer (UV 2550, Shimadzu). 

## 3. Results and Discussion

**Figure 1:**
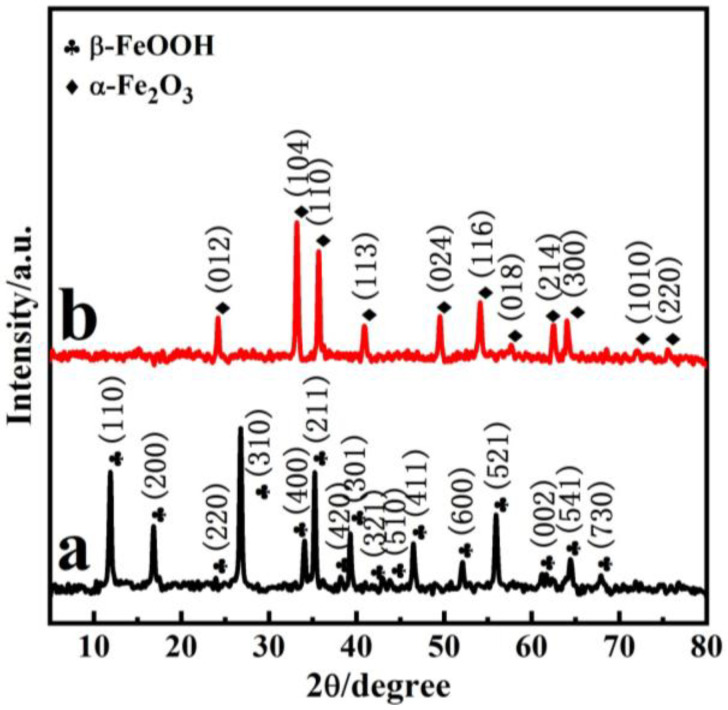
XRD patterns of the precursor (**a**) and as-prepared α-Fe_2_O_3_ (**b**).

Figure 1 demonstrates the typical XRD patterns of the precursor β-FeOOH (P85) and α-Fe_2_O_3_ (P85-1). From Figure 1a, there are peaks at 11.83°, 16.74° and 26.85°, and so on, which are in good agreement with the JCPDS file of β-FeOOH (JCPDS 34-1266) [36,37]. As is shown in Figure 1b, the diffraction peaks at 24.0°, 33.0°, 35.5°, 40.7°, 49.3°, 54.0°, 57.6°, 62.3°, 63.9°, 71.8° and 75.3° were attributed to (012), (104), (110), (113), (024), (116), (018), (214), (300), (1010) and (220) facets of α-Fe_2_O_3_ nanopolyhedrons, respectively, which is consistent with the JCPDS file of α-Fe_2_O_3_ (JCPDS 33-0664) [27,38]; this is consistent with the XRD results of commercial Fe_2_O_3_ nanoparticles (Figure S1 in Supplementary Materials). In addition, characteristic peaks of impurities could not be observed; this indicates the phase transition from β-FeOOH to α-Fe_2_O_3_. The augmented peak sharpness in Figure 1b indicates that α-Fe_2_O_3_ is well crystallized. 

**Figure 2:**
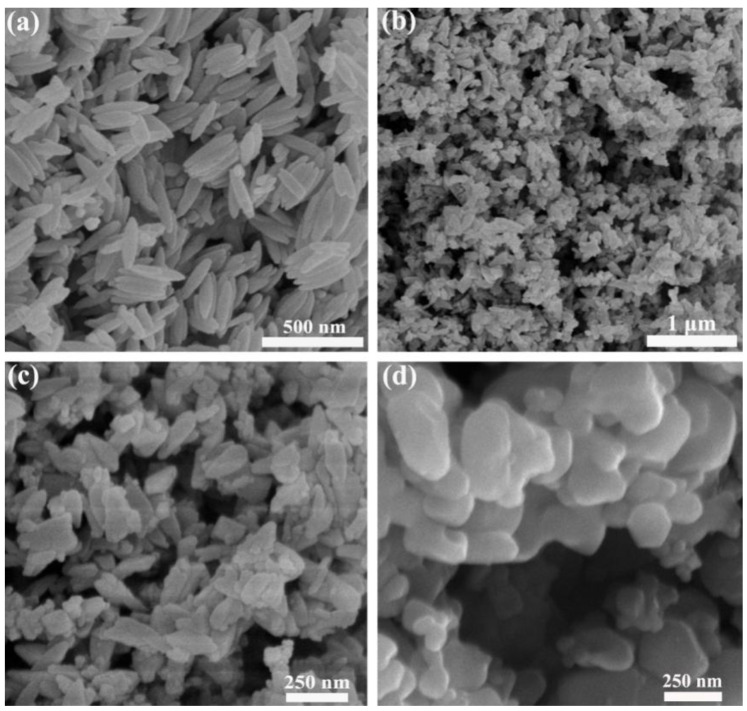
SEM images of (**a**) β-FeOOH, (**b**,**c**) α-Fe_2_O_3_ and (**d**) commercial Fe_2_O_3_ nanoparticles.

The morphology of the samples was detected by SEM. Figure 2 demonstrates the SEM images of the precursor P85, sample P85-1 and commercial Fe_2_O_3_ nanoparticles. Figure 2a and Figure S2 (Supplementary Materials) show the images of the precursor, which clearly demonstrate that the nanorods were of a length of about 200 nm and a diameter of about 60 nm. The surface of the nanorods was smooth and the smooth-surfaced particles were similar in size. Figure 2b,c shows the images of P85-1, which had changed following sintering, from a fusiform shape to irregular rods. The rod-shaped particles were polymerized, with a length of about 125 nm and a diameter of 50 nm. Figure 2d shows the images of commercial Fe_2_O_3_ nanoparticles were near-spherical. It can be concluded from Figure 2 that Fe_2_O_3_ nanoparticles with different morphologies were prepared under different experimental conditions, and the prepared Fe_2_O_3_ exhibited a more regular fusiform-like structure.

**Figure 3:**
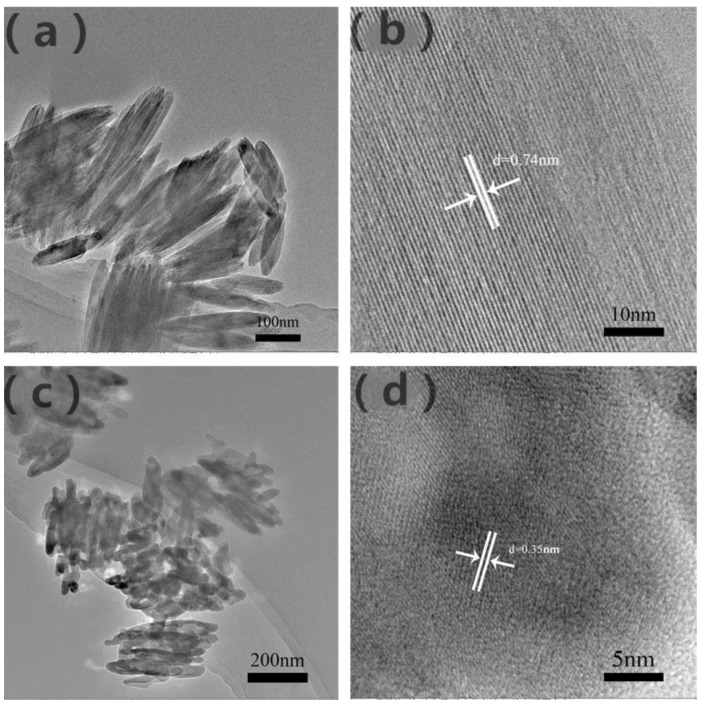
TEM images of β-FeOOH (**a**) and (**b**), α-Fe_2_O_3_ (**c**) and (**d**).

The size and microstructure of the prepared β-FeOOH and α-Fe_2_O_3_ samples were further examined with TEM in Figure 3a,c. Furthermore, Figure 3b demonstrates a lattice fringe of 0.74 nm corresponding to the (110) facet of β-FeOOH, which further confirmed that P85 is β-FeOOH. Besides this, the distance of 0.35 nm of α-Fe_2_O_3_ could clearly identify lattice spacing, which corresponds to interplane distances of (012) plane in Figure 3d, consistent with the XRD results [27,38].

**Figure 4:**
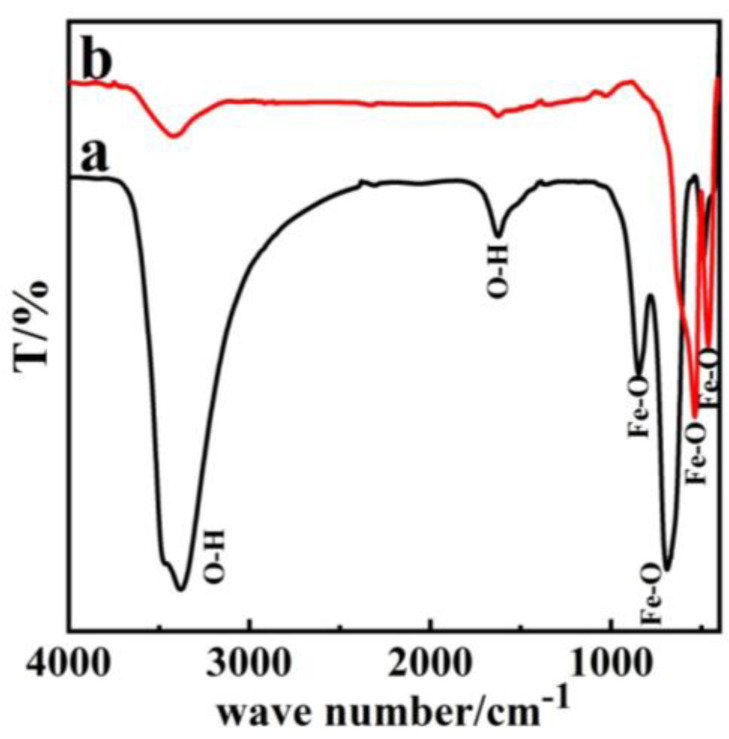
FT-IR image of (**a**) β-FeOOH (**b**) α-Fe_2_O_3_.

The infrared spectrum of β-FeOOH in Figure 4a demonstrates that the precursor P85 showed absorption peaks at 3440 cm^−1^ and 1600 cm^−1^, 850 cm^−1^, 700 cm^−1^. Of these, the peaks at 3440 cm^−1^ and 700 cm^−1^ showed strong absorption, and the peak at 3440 cm^−1^ corresponded to symmetric and anti-symmetric stretching vibrations of O–H group. The peak at 1600 cm^−1^ corresponded to the bending vibration of O–H bond, while the other two peaks at 850 cm^−1^ and 700 cm^−1^ corresponded to the stretching vibration of Fe–O bond [30,38]. Figure 4b demonstrated that the strong absorption peaks of α-Fe_2_O_3_ only emerge at 508 cm^−1^ and 480 cm^−1^. In addition, the two stretching vibration peaks for Fe–O at 3440 cm^−1^ and 1600 cm^−1^ only appeared as weak absorption peaks, which is consistent with commercial Fe_2_O_3_ results (Figure S3 in Supplementary Materials). This is because the O–H bond absorption had disappeared completely, which indicated that the precursor β-FeOOH had transformed to α-Fe_2_O_3_ [23,38,39].

**Figure 5:**
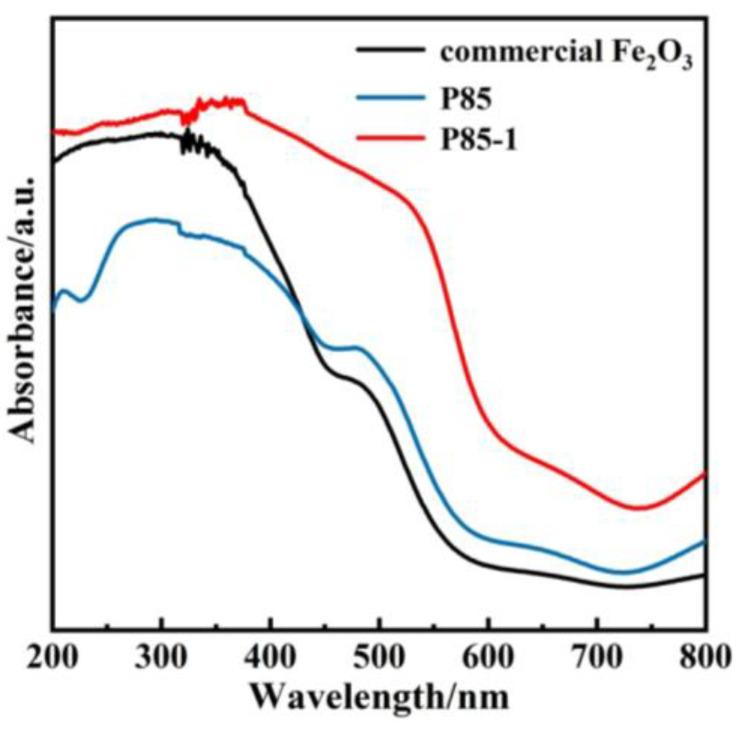
UV–Vis diffuse reflectance of P85, P85-1 and commercial Fe_2_O_3_.

UV–Vis diffuse reflectance was measured through ultraviolet and visible light absorption technology. It can be seen from Figure 5 that the commercial Fe_2_O_3_ showed a narrow absorption of visible light with an edge that occurred at around 450 nm. However, compared with the commercial Fe_2_O_3_ and the P85 precursor, the photo absorption edge of the prepared α-Fe_2_O_3_ nanorods showed a more obvious redshift from 450 nm to 550 nm, so that the optical absorption of α-Fe_2_O_3_ nanorods was significantly stronger in visible-light regions [4,10,40]. Furthermore, the increase of light absorption range was conducive to the enhancement of photocatalytic activity. In addition, the UV–Vis diffuse reflectance was further combined and the band gap diagram was calculated via Tauc plot (Figure S4 in Supplementary Materials). Compared with the band gap of commercial Fe_2_O_3_ (2.2 eV), the band gap (1.95 eV) of P85-1 was significantly shorter, indicating that P85-1 has a stronger light utilization efficiency and enhanced photocatalytic ability.

**Figure 6:**
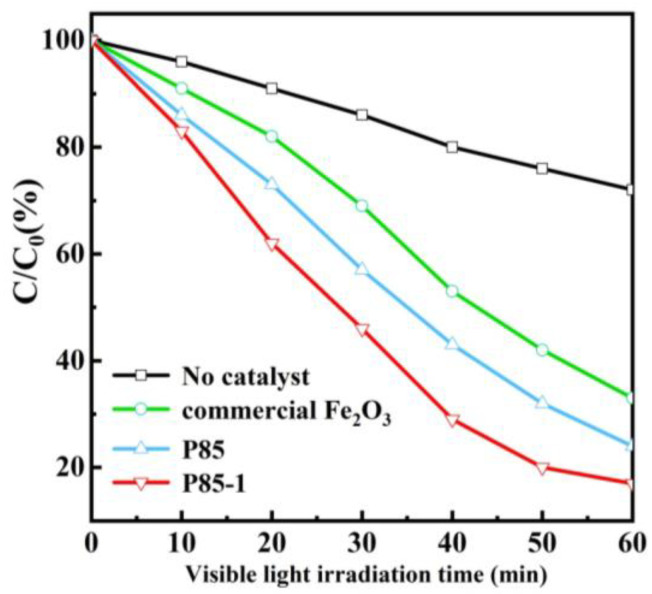
Photocatalytic degradation of methylene blue (MB) solution (40 mL, 10 mg/L) with no catalyst, P85, P85-1, and commercial Fe_2_O_3_ under visible light irradiation (λ > 410 nm).

Methylene blue (MB) is a highly significant dye and has been extensively applied in industrial production, which inevitably pollutes the environment. Therefore, the photocatalytic performance of prepared α-Fe_2_O_3_ was evaluated by degradation of MB under visible light irradiation. Before the photocatalytic process, the solution containing MB and catalysts were agitated to reach the adsorption equilibrium in the dark. Figure 6 demonstrates the photocatalytic evaluation curve of the prepared α-Fe_2_O_3_ under visible light. The figure clearly shows the variation of MB solution concentrations with degradation time and the fitting result of degradation kinetics. With the extension of light time, the MB solution concentration gradually decreased and the degradation process conformed to a first-order kinetic model. After 60 min of light, the degradation rate of MB of α-Fe_2_O_3_ reached 83%, which is better than that of commercial Fe_2_O_3_ and the P85 precursor. Moreover, the catalyst-free condition was also used as a comparative experiment and its degradation rate was only about 25%. This further confirms that the prepared α-Fe_2_O_3_ exhibits remarkably high photocatalytic activity, which is consistent with the results on photocurrent (Figure S5 in Supplementary Materials). The repeated degradation experiments of α-Fe_2_O_3_ (P85-1) showed a slight decrease in MB degradation rate after four cycles, indicating that it had high stability (Figure S6 in Supplementary Materials). Besides this, there were no significant differences in the XRD spectra of α-Fe_2_O_3_ before and after four cycles of use, which further proves its stability (Figure S7 in Supplementary Materials).

## 4. Conclusions

In conclusion, a simple one-step hydrothermal method was used to prepare three-dimensional fusiform-like α-Fe_2_O_3_, which was applied to photocatalytic degradation of MB. It could be seen through SEM and TEM that the three-dimensional fusiform-like α-Fe_2_O_3_ was about 125 nm in length and 50 nm in diameter. In addition, the prepared α-Fe_2_O_3_ showed excellent degradation efficiency of MB solution under visible light illumination, which reached 83% after 60 min sunlight illumination. This is far superior to traditional commercial Fe_2_O_3_ and further proves α-Fe_2_O_3_’s excellent photocatalysis performance. As a result, α-Fe_2_O_3_ has a wider visible light absorption edge, which promotes the improvement of photocatalytic activity, thereby showing better degradation performance. Furthermore, the as-prepared samples exhibited more advantages such as being low cost, environmentally friendly and low risk. Therefore, this work provides a promising and constructive theoretical support for solving problems such as energy shortages and environmental pollution in the future.

## Data Availability

The data presented in this study are available on request from the corresponding author.

## Supplementary Materials

The following are available online at https://www.mdpi.com/article/10.3390/nano11102650/s1, Figure S1: XRD pattern of the commercial Fe_2_O_3_; Figure S2: SEM images of β-FeOOH; Figure S3: FT-IR image of the commercial Fe_2_O_3_; Figure S4: The band gaps of P85-1 and commercial Fe_2_O_3_ determined from Tauc plots; Figure S5: IT-curves of P85-1; Figure S6: Cycle stability experiment of P85-1; Figure S7: XRD pattern of P85-1 after four cycle times.
