# Supplementary material for: Facile Fabrication of Three-Dimensional Fusiform-Like α-Fe2O3 for Enhanced Photocatalytic Performance
Source: Nanomaterials (Basel). 2021 Oct 9;11(10):2650. doi: 10.3390/nano11102650 (PMC8539989; doi:10.3390/nano11102650)
Supplement: Supplementary file 1 [file nanomaterials-11-02650-s001.zip › nanomaterials-1384752-supplementary.pdf]

Supporting Materials

Facile Fabrication of Three-Dimensional Fusiform-like α-Fe_2O_3 for Enhanced Photocatalytic Performance

Moyan Li [1,2,3], Hongjin Liu [1], Shaozhi Pang [1], Pengwei Yan [1], Mingyang Liu [1,*], Minghui Ding [1,2,3,*] and Bin Zhang [1,2,3]

1 College of Material Science and Chemical Engineering, Harbin Engineering University, Harbin 150001, China; lmy604333@163.com (M.L.); b9838@126.com (H.L.); 18332768710@163.com (S.P.); yanpengwei@hrbeu.edu.cn (P.Y.); zhangbin_hipc@126.com (B.Z.)
2 Key Laboratory of Super Light Material and Surface Technology, Ministry of Education, Harbin Engineering University, Harbin 150001, China
3 Institute of Surface/Interface Science and Technology, Harbin Engineering University, Harbin 150001, China
* Correspondence: lmy_0202@163.com (M.L.); ding197392@163.com (M.D.); Tel./Fax: +86-451-8251-8219

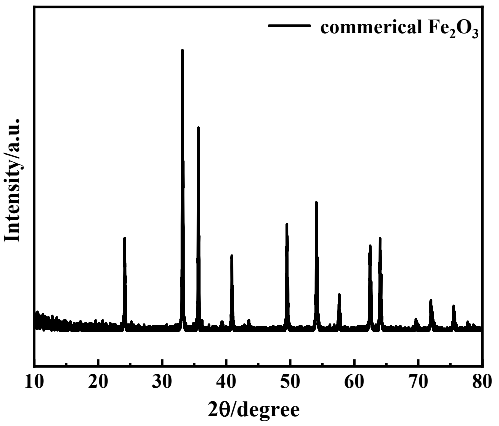

Figure S1. XRD pattern of the commercial Fe_2O_3.

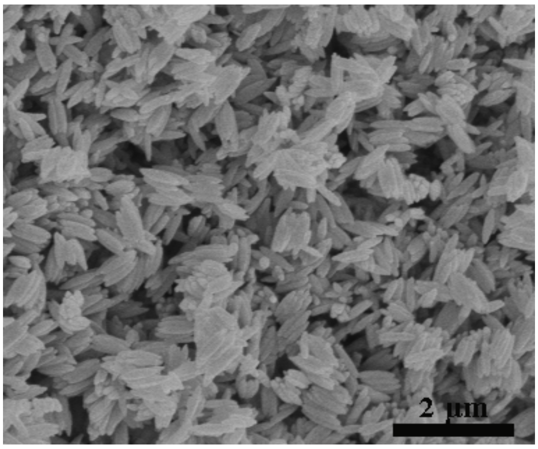

Figure S2. SEM images of β-FeOOH

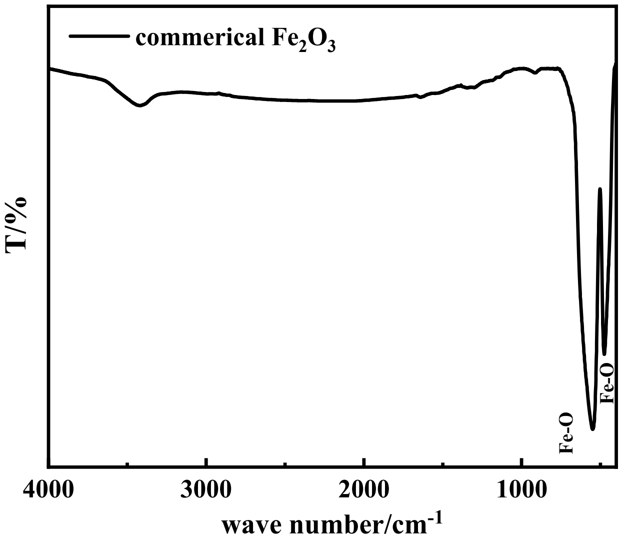

Figure S3. FT-IR image of the commercial Fe_2O_3.

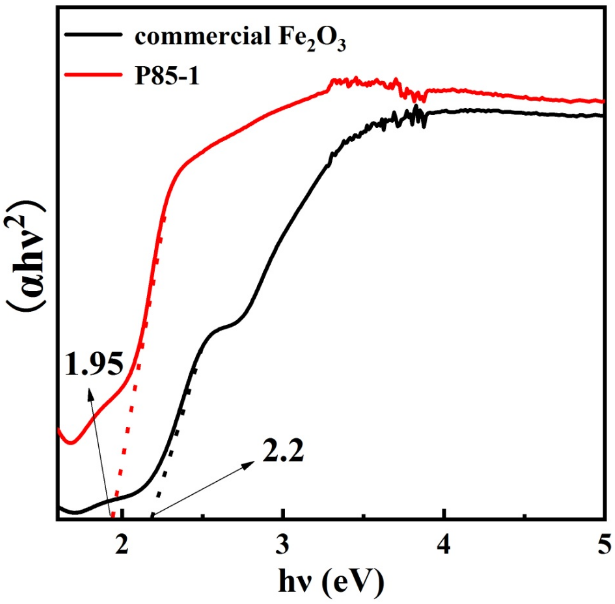

Figure S4. The band gaps of P85-1 and commercial Fe_2O_3 determined from Tauc plots.

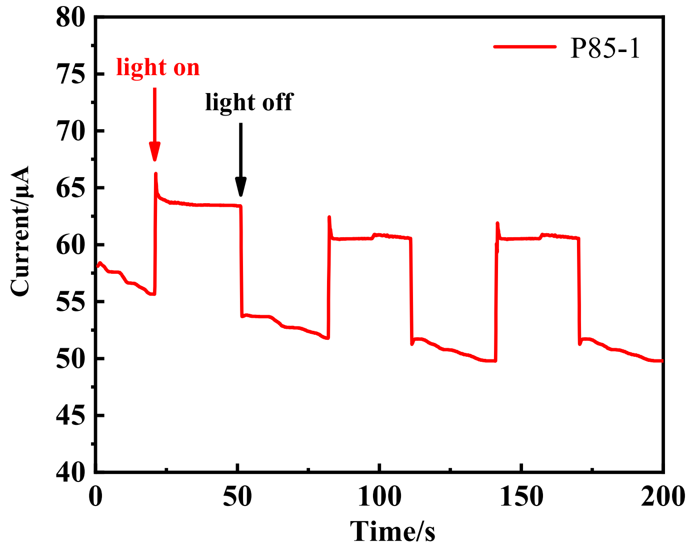

Figure S5. IT-curves of P85-1

It shows the transient photocurrent intensity of prepared samples. Specifically, the α-Fe_2O_3 photocatalyst shows a high photocurrent intensity, which provides better condition of separating photo-induced carriers. The above conclusions indicate that α-Fe_2O_3 photocatalyst can efficiently separate photogenerated charges and it has the best photocatalytic activity.

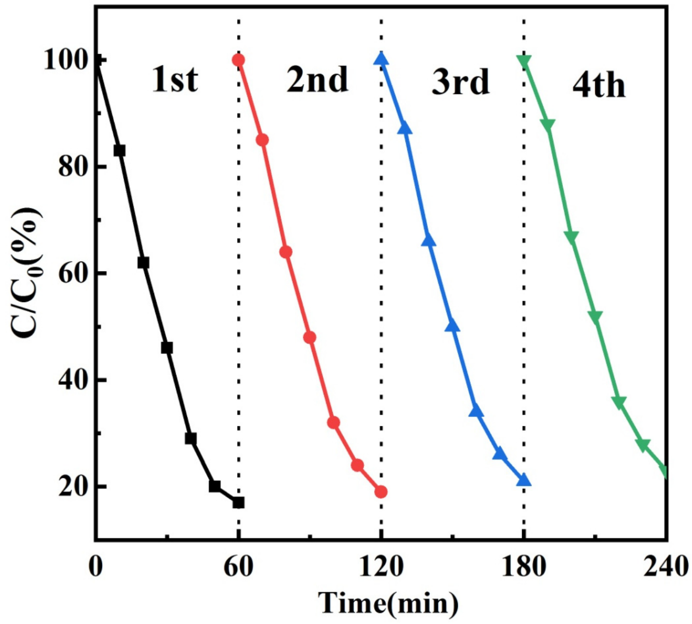

Figure S6. Cycle stability experiment of P85-1.

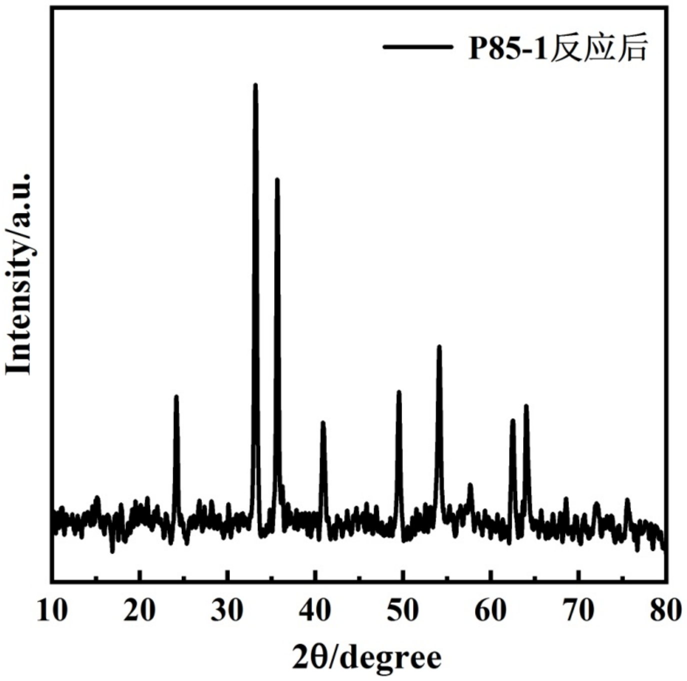

Figure S7.XRD pattern of P85-1 after four cycle times.
